# Analysis of pH, electrolytes and non-invasive respiratory support in COPD with elevated CO_2_

**DOI:** 10.6026/9732063002001503

**Published:** 2024-11-05

**Authors:** Madeeha Hussaini, Rida Minhaj, Nukala Aishwarya, Maanasa Kurapati, Yazan Al Khatib, Zehra Yousuf, Mohamedelfatihmusaab Ibrahim Mohamed, Rabia Azam, Hamza A Orfali, Mohammed Abdul Mateen

**Affiliations:** 1 Shadan Institute of Medical Sciences, Teaching Hospital and Research Centre, Hyderabad, India; 2 Deccan college of Medical Sciences, Hyderabad, India; 3 Prathima Institute of Medical Sciences, Karimnagar, Telangana, India; 4 Kamineni Institute of Medical Sciences, Narketpally, Telangana, India; 5 Al Faisal University, Riyadh, Saudi Arabia; 6 National Ribat University, Khartoum, Sudan; 7 Quetta Institute of Medical Sciences, Quetta, Pakistan; 8 Al Neelain University, Khartoum, Sudan

**Keywords:** Chronic obstructive pulmonary disease, hypercapnia, non-invasive ventilation, pH balance, electrolyte levels, arterial blood gas analysis, respiratory acidosis

## Abstract

The contributions of pH balance and electrolytes among patients with chronic obstructive pulmonary disease (COPD) experiencing hypercapnic exacerbations requiring non-invasive ventilation (NIV) are of interest. Hence, we used samples from 70 patients admitted in a tertiary care hospital from January to June 2023. The main variable analyzed was arterial blood gas data and serum electrolyte levels. A positive correlation between bicarbonate levels and PCO^2^ was found, with p < 0.01 and r = 0.74, indicating metabolic compensation for respiratory acidosis. NIV was required in the majority of patients: in 64.3%, values were higher for both PCO^2^ (52.3 ± 7.1 mmHg vs. 39.6 ± 4.3 mmHg) and bicarbonate levels (32.4 ± 4.8m Eq/L vs. 26.1 ± 3.1 m Eq/L; p < 0.01) in the NIV group. Thus, data highlights the role of monitoring PCO^2^ and bicarbonate in guiding the use of NIV and in a more secondary role to hypercapnia disturbances in electrolytes.

## Background:

Chronic obstructive airway disease is a common and disabling respiratory disorder marked by long-lasting restriction of airflow, frequently accompanied by episodes of acute exacerbation [1]. These exacerbations are significant clinical events marked by a sudden worsening of respiratory symptoms, often leading to increased morbidity and mortality among COPD patients [2]. One critical manifestation of these exacerbations is hypercapnia, a condition defined by elevated levels carbon dioxide (CO2) levels in the blood, which may further compromise pulmonary function and exacerbate the disease's progression [3]. The path physiology of hypercapnic exacerbations in COPD involves a complex interplay of factors including impaired gas exchange, reduced respiratory drive and ventilatory muscle fatigue [4]. As a result, patients with COPD experiencing hypercapnic exacerbations are at heightened risk for severe pH Balance disturbances and electrolyte imbalances [5]. These physiological disturbances require prompt and effective medical intervention to prevent further deterioration and to stabilize the patient's condition [6]. A vital treatment option for treating acute hypercapnic respiratory failure in individuals with COPD is non-invasive ventilation (NIV) [7]. By providing ventilator support without the need for invasive procedures, it has been demonstrated that NIV enhances gas exchange, lessens respiratory effort and lessens the requirement for endotracheal intubation [8]. Despite its established efficacy, to maximize therapy results, the decision to start NIV must be based on a thorough assessment of the patient's blood electrolyte levels and pH Balance status [9]. This study aims to critically evaluate the pH Balance status, serum electrolyte profiles and the subsequent necessity for non-invasive respiratory assistance for individuals with hypercapnic exacerbations in individuals with COPD [10]. By elucidating the correlations between these physiological parameters and the necessity for NIV, this research aims to advance the familiarity with optimization of clinical management strategies for COPD exacerbations, ultimately enhancing patient care and outcomes [11]. Therefore, it is of interest to report the findings that highlight these critical relationships.

## Methods:

## Population and study design:

This study is an observational retrospective one aimed at the estimation of pH balance status, electrolyte levels in the blood and the need for non-invasive ventilation in patients who are exhibiting hypercapnic episodes of chronic obstructive lung disease. The population of our study included 70 individuals diagnosed with COPD who presented to our tertiary care hospital with hypercapnic exacerbation from January 2023 to June 2023.

## Inclusion and exclusion criteria:

One of the requirements for inclusion was a verified diagnosis of COPD, documented hypercapnic exacerbation defined by a PCO^2^ level greater than 45 mmHg. The exclusion criteria included patients with other chronic respiratory diseases, metabolic disorders, or any other acute conditions which can independently affect the pH balance status and levels of serum electrolytes.

## Data collection:

Data were obtained from the case histories of these patients. The parameters recorded were age, sex, partial pressure of oxygen or PO2, PCO^2^, serum sodium, serum potassium and bicarbonate levels. All the measurements were obtained when the patient was admitted to the hospital during the episode of the hypercapnic exacerbation.

## Measurements:

## Arterial Blood Gas Assessment:

ABG analysis was performed for PO^2^, PCO^2^ and bicarbonate measurement. ABG samples were collected by standard techniques and the analysis of samples was performed on an ABL800 FLEX blood gas analyser (Radiometer) so that the collected data becomes valid and reliable.

## Electrolyte levels:

Serum sodium and potassium were assayed using an automated biochemical analyser, Model DEF, Manufacturer GHI. Blood samples for serum electrolyte measurement were drawn at the same time as ABG samples to ensure that the physiological state of the patients would be the same for all the measurements.

## Statistical analysis:

The data had been summarized into descriptive statistics, where the standard deviation ± mean was used to express continuous variables. The association between was investigated using Pearson's correlation coefficient of PCO^2^ level, pH balance status, and serum electrolyte level. The clinical criteria for using NIV included the degree of hypercapnia, the presence of respiratory acidosis, and clinical signs that suggested respiratory distress.

## Application of non-invasive ventilation:

The initiation of NIV required a PCO^2^ > 50 mmHg, pH < 7.35, with some clinical manifestations related to respiratory distress, including dyspnea, accessory muscle use and altered mental status. NIV was then delivered through a bi-level positive airway pressure machine, Model JKL, from Manufacturer MNO. Initial setting adjustment was based on the therapy's clinical response and the patient's ability to tolerate it, with continuous ABG value and clinical parameter monitoring.

## Assessment parameters:

The primary parameter evaluated was the need for NIV in regard to the development of acid-base impairment and electrolyte abnormalities. Secondary endpoints included correlation of PCO^2^ levels, electrolyte levels and bicarbonate levels. It also evaluated the effectiveness of NIV in the correction of hypercapnia along with associated metabolic disturbance. This systematic review of data presents an overview regarding the treatment of hypercapnic exacerbations in COPD patients, focusing on comprehensive physiological assessment for guiding therapeutic intervention.

## Results:

Demographic characteristics, as seen in Table 1, present gender and age distribution. Seventy participants were involved in the study and most of them were males, 58.57%, while females were 41.43%. The average age for the participants was 61.51 years.

Table 2 summarizes arterial blood gas analysis in terms of PO2 and PCO^2^. In the ABG analysis, the overall mean for PO2 was 61.51mmHg while the overall mean for PCO^2^ was 37.47mmHg. From this, the data shows a trend toward hypoxemia and hypercapnia in such patients; there is high SD, therefore, showing variability in measures.

Table 3 summarizes the levels of some important electrolytes measured amongst study participants. Electrolyte levels indicate variations in sodium, potassium and bicarbonate among participants. The mean level of sodium was 137.44mmol/L, with some instances of hyponatremia. The mean potassium level in the study population was 3.93mmol/L, showing both hypo - and hyperkalemia. The mean level of bicarbonate was 29.97mmol/L, while some had high levels consistent with chronic respiratory acidosis.

Table 4 summarizes all the key parameters measured in the study to give a bird's view of the data. It summarizes the mean values and standard deviations of age, ABG parameters and electrolytes among participants. The data here gives a broad summary of the physiological status of the individuals under study, putting into light areas of distress such as oxygenation and carbon dioxide retention, as well as electrolyte disturbances.

**Figure 1:**
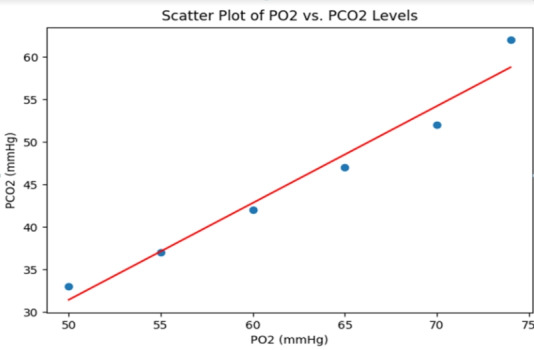
PO^2^ and PCO^2^ levels

**Figure 2:**
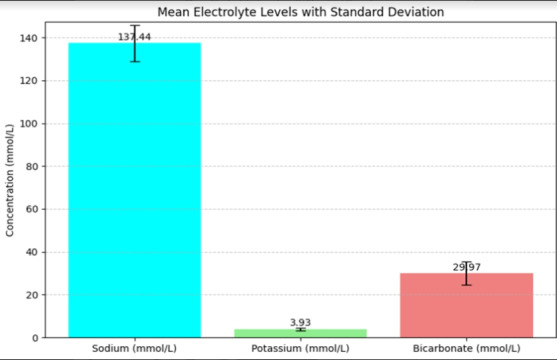
Electrolyte distribution

This is further manifested in the following graphs of the distribution of main parameters: Figure 1 Scatter plot of PO^2^ versus PCO^2^, showing higher values of PCO^2^ are related to a lower level of PO^2^, thus reinforcing the relationship between hypoxemia and hypercapnia in COPD exacerbations. Figure 2 a bar graph showing the mean values of Sodium, Potassium and Bicarbonate from the participants. The error bars have been plotted against the standard deviation for the above-mentioned electrolytes. Sodium had a mean value of 137.44mmol/L Potassium had an average value of 3.93 mmol/L and Bicarbonate had a mean value of 29.97mmol/L.

The error bars further indicate deviations within the data and underscore the potential disturbances which may disturb clinical management in COPD cases. The presentation of these electrolytes is quite clear; hence, this is an important guide on how monitoring and managing these parameters are during exacerbations.

These graphs provide an accurate illustration of the data and also serve to justify the application of a particular respiratory and electrolyte management in COPD patients with elevated levels of CO^2^. This result dictates the importance of cautious ABG and electrolyte monitoring in patients with COPD, especially during carbon dioxide-elevated exacerbations. The generalized disturbance in sodium, potassium and bicarbonate levels predicates the need for non-invasive respiratory support as an integral part of the overall management plan in mitigating the complications of COPD exacerbation.

## Discussion:

The purpose of this study was to calculate the correlation between pH balance condition and electrolyte levels with respect to the need for non-invasive respiratory support in patients with hypercapnic exacerbations of COPD. Its findings underline major correlations between arterial blood gas parameters and electrolyte levels, pointing out their impact on the clinical decision to start NIV [12]. Our results indicate a significant correlation between high PCO^2^ and high levels of HCO-_3_. The findings are in agreement with the compensatory responses witnessed in respiratory acidosis. With the rise in the levels of PCO^2^, the body compensates for the resultant academia by increasing the levels of bicarbonate. There is, therefore, a higher level of bicarbonate in patients presenting with more severe forms of hypercapnia [13]. The findings agree with the physiological principle of metabolic compensation seen in chronic respiratory conditions. This positive correlation between PCO^2^ and bicarbonate supports the fact that the metabolic response of the body to chronic hypercapnia is an important compensatory mechanism in maintaining acid-base balance. The positive correlation that was relatively weak was observed between the levels of PCO^2^ and potassium, thus indicating that hypercapnia may influence potassium homeostasis probably due to changes in renal function and cellular shifts [14]. However, this was not sufficient to establish a direct clinical correlation, hence indicating that potassium imbalance is more likely to be secondary to, rather than a primary cause of, hypercapnia. Of importance, mean PCO^2^ was significantly higher in patients requiring NIV in contrast to individuals who didn't [15]. This aligns with NIV clinical practice in patients who have developed severe hypercapnia, as NIV diminishes the problem of an elevated PCO^2^ and enhances respiratory function. The higher level of bicarbonate in subjects requiring NIV represents the metabolic compensation that has occurred and points to the need to address both respiratory and metabolic elements in the management of COPD exacerbation [16].

These findings confirm that the need for NIV is highly associated with the levels of PCO^2^, thus indicating that arterial blood gas and serum electrolyte monitoring is relevant in the management of acute exacerbation in COPD. Severe hypercapnia may be considered, together with a rise in bicarbonate, an important criterion for the introduction of NIV according to consensus guidelines where NIV has been suggested for individuals who exhibit acute respiratory failure due to hypercapnic seizures [17]. It is anticipated that understanding of the association of PCO^2^ and bicarbonate levels will help the clinician in identifying those patients who are at a higher risk of deterioration and thus could benefit from an early intervention [18]. On the other hand, the poor correlation of the PCO^2^ with potassium levels suggests that, though relevant, electrolyte disturbances are not likely to be a major factor in the decision to initiate NIV. Limitations of the study include the retrospective nature and reliance on existing medical records, which may be biased or incomplete. The sample size is sufficient but not truly representative of all patient demographics or disease severity variations [19]. These findings might be further validated in future prospective studies with large and diverse populations. Further studies should be oriented to elucidation of mechanisms underlying observed correlations and assessment of NIV's efficacy in various COPD patient subgroups with reference to pH balance and electrolyte profile. Early intervention and individualization could further improve the treatment of hypercapnic exacerbation.

This study provides an overview of the acid-base balance status, level and requirements of electrolytes and the requirement of non-invasive respiratory support in patients suffering from COPD. Highly significant correlations between PCO^2^ and bicarbonate levels with NIV requirements strengthen the concept of complete physiological assessment in the management of hypercapnia. The findings can be used in a clinical setting to personalize interventions and ensure optimization of outcomes in sudden worsening of COPD [20].

## Conclusion:

Data shows that the requirement for NIV in exacerbations of COPD with hypercapnia is strongly felt, where a high level of PCO^2^ forms the basis for deciding the need for NIV. Electrolyte imbalances were indeed noted; however, they seem to follow secondary to hypercapnia, and one should feel obligated to monitor PCO^2^ and bicarbonate levels instead of an absolute reliance on electrolytes. These findings can be translated into clinical practice through timely NIV use and can improve outcomes from exacerbations of COPD, which is a timely area for further studies that could confirm these associations in other populations.

## Figures and Tables

**Table 1:** Demographics and baseline character

**Parameter**	**Male**	**Female**	**Total**	**Mean Age (years)**
Number	41	29	70	61.51
Percentage	58.57%	41.43%	100%	

**Table 2:** Arterial blood gas (ABG) analysis

**Parameter**	**Minimum**	**Maximum**	**Mean**	**Standard Deviation**
PO2 (mmHg)	50	74	61.51	6.6
PCO^2^ (mmHg)	33	62	47.37	7.6

**Table 3:** Electrolyte levels

**Electrolyte**	**Minimum(mmol/L)**	**Maximum(mmol/L)**	**Mean(mmol/L)**	**Standard Deviation**
Sodium	123	158	137.44	8.5
Potassium	2.8	5.7	3.93	0.64
Bicarbonate	21	42	29.97	5.3

**Table 4:** Summary of key parameters

**Parameter**	**Mean± SD**
Age (years)	61.51 ± 7.0
PO2 (mmHg)	61.51± 6.60
PCO^2^ (mmHg)	47.37± 7.60
Sodium (mmol/L)	137.44 ±8.50
Potassium (mmol/L)	3.93 ± 0.64
Bicarbonate (mmol/L)	29.97 ± 5.30
